# Expression of Wild-Type and Mutant Human TDP-43 in Yeast Inhibits TOROID (TORC1 Organized in Inhibited Domain) Formation and Autophagy Proportionally to the Levels of TDP-43 Toxicity

**DOI:** 10.3390/ijms25116258

**Published:** 2024-06-06

**Authors:** Sangeun Park, Sei-Kyoung Park, Susan W. Liebman

**Affiliations:** Department of Pharmacology, University of Nevada, Reno, NV 89557, USA

**Keywords:** autophagy, TOROID, TDP-43, TORC1, *TOR1*, *PBP1*, *TIP41*, yeast

## Abstract

TDP-43 forms aggregates in the neurons of patients with several neurodegenerative diseases. Human TDP-43 also aggregates and is toxic in yeast. Here, we used a yeast model to investigate (1) the nature of TDP-43 aggregates and (2) the mechanism of TDP-43 toxicity. Thioflavin T, which stains amyloid but not wild-type TDP-43 aggregates, also did not stain mutant TDP-43 aggregates made from TDP-43 with intragenic mutations that increase or decrease its toxicity. However, 1,6-hexanediol, which dissolves liquid droplets, dissolved wild-type or mutant TDP-43 aggregates. To investigate the mechanism of TDP-43 toxicity, the effects of TDP-43 mutations on the autophagy of the GFP-ATG8 reporter were examined. Mutations in TDP-43 that enhance its toxicity, but not mutations that reduce its toxicity, caused a larger reduction in autophagy. TOROID formation, which enhances autophagy, was scored as GFP-TOR1 aggregation. TDP-43 inhibited TOROID formation. TORC1 bound to both toxic and non-toxic TDP-43, and to TDP-43, with reduced toxicity due to *pbp1Δ*. However, extragenic modifiers and TDP-43 mutants that reduced TDP-43 toxicity, but not TDP-43 mutants that enhanced toxicity, restored TOROID formation. This is consistent with the hypothesis that TDP-43 is toxic in yeast because it reduces TOROID formation, causing the inhibition of autophagy. Whether TDP-43 exerts a similar effect in higher cells remains to be determined.

## 1. Introduction

Yeast has been a useful model to investigate human proteins that are associated with neurodegenerative diseases and form amyloid-like aggregates [1,2,3,4,5,6,7]. Here, we study TDP-43 (TAR DNA-binding protein 43), which often aggregates in the neurons of patients with ALS (amyotrophic lateral sclerosis) and other diseases [8]. In yeast, when TDP-43 is expressed, it forms aggregates and is toxic [2]. Wild-type TDP-43 must be expressed more than TDP-43 mutants that cause familial ALS to achieve the same level of toxicity [8,9]. Since yeast normally lacks TDP-43, yeast cells cannot detect toxicity due to the inhibition of TDP-43 function, although this does contribute to its harmful effects in mammalian cells [10,11]. Thus, toxicity caused by the presence of TDP-43 in yeast is a good model for toxicity that occurs specifically due to a toxic form of TDP-43 in human cells.

Genetic screens in yeast have identified proteins that, when not expressed or when overexpressed, alter TDP-43 toxicity [2,12,13,14,15,16]. One such modifier is *PBP1* (poly (A) binding protein). Finding, in yeast, that the level of *PBP1* modifies TDP-43 toxicity led to the discoveries that alterations in *PBP1*’s human homolog, *ATXN2* (ataxin-2), increase the human risk for ALS, and that lowering the level of *ATXN2* reduces the toxicity of TDP-43 in mice [17,18,19,20]. This shows that the yeast model is relevant to human diseases associated with TDP-43.

TDP-43 as well as other RNA-binding proteins separate into membrane-less structures, e.g., stress granules, during their normal function in cells. These membrane-less organelles (also called liquid-like droplets) form via liquid–liquid phase separation within the cytoplasm and are dynamic [21]. Liquid-like droplets formed in vitro by proteins such as FUS (fused in sarcoma), hnRNPA1 (heterogeneous nuclear ribonucleoprotein A1), and TDP-43 LCD (low-complexity domain) have been shown to turn into non-dynamic amyloid fibrils [22,23,24,25]. Amyloids are highly ordered protein aggregates in a cross-β sheet structure that thioflavin T will stain [26].

The structure of TDP-43 aggregates found in patients or formed in vitro remains under dispute [27]. Some find that the low-complexity TDP-43 C-terminal domain forms amyloid in vitro [28], and others that neither in vitro-formed TDP-43 filaments (full-length or C-terminal) nor TDP-43 aggregates formed in situ in neurons have amyloid properties [27,29]. Although TDP-43 aggregates are associated with disease, it is not known if they cause the disease or are a consequence of the disease [30]. Indeed, a recent study of mutations in the prion-like domain of TDP-43 that enhance or reduce TDP-43 toxicity in yeast suggests that solid aggregates might be protective [31]. Mutations that enhanced toxicity decreased the hydrophobicity of TDP-43 and enhanced the formation of small TDP-43 foci near the nucleus. In contrast, mutations that reduced TDP-43 toxicity increased TDP-43 hydrophobicity and promoted the formation of larger aggregates that were primarily in the cytoplasm. FRAP (fluorescence recovery after photobleaching) measurements showed that the large cytoplasmic TDP-43 foci were solid aggregates, while the TDP-43 in the small nuclear-associated foci was dynamic. Thus, the authors proposed that the small foci are liquid-like and that they are more toxic than the solid cytoplasmic TDP-43 aggregates [31]. Here, we investigate the structure of TDP-43 aggregates in yeast. We test if the dynamic TDP-43 aggregates made by toxic TDP-43 mutants are liquid-like and if the solid TDP-43 aggregates made by less toxic TDP-43 mutants are amyloid.

The mechanism by which TDP-43 causes toxicity is unknown. However, the expression of TDP-43 reduces autophagy in yeast, and *pbp1-Δ* and *tip41-Δ* (target of rapamycin signaling pathway inhibitor protein 41) deletions, which lower TDP-43 toxicity, reverse TDP-43’s effect on autophagy [32]. While we do not know how TDP-43 affects autophagy, one possibility is that TDP-43’s effect on toxicity and autophagy is due to an effect on TORC1 (target of rapamycin complex 1). TORC1 inhibits autophagy [33] when it is located at the vacuolar membrane in yeast. TOROIDs (TORC1 organized in inhibited domains) are large helical polymers of TORC1 condensed in foci that are associated with the vacuole. When TORC1 condenses into a TOROID, TORC1’s inhibition of autophagy is reduced. TOROID formation is known to be triggered by glucose starvation [34]. Here, we ask if TDP-43 can inhibit TOROID formation and test if TDP-43 toxicity is correlated with (1) its effects on autophagy, (2) its effects on TOROID formation, or (3) its binding to TORC1.

## 2. Results

### 2.1. Probing the Structure of TDP-43 Aggregates: Analysis of Thioflavin T Binding and Sensitivity to 1,6-Hexanediol

**Figure 1:**
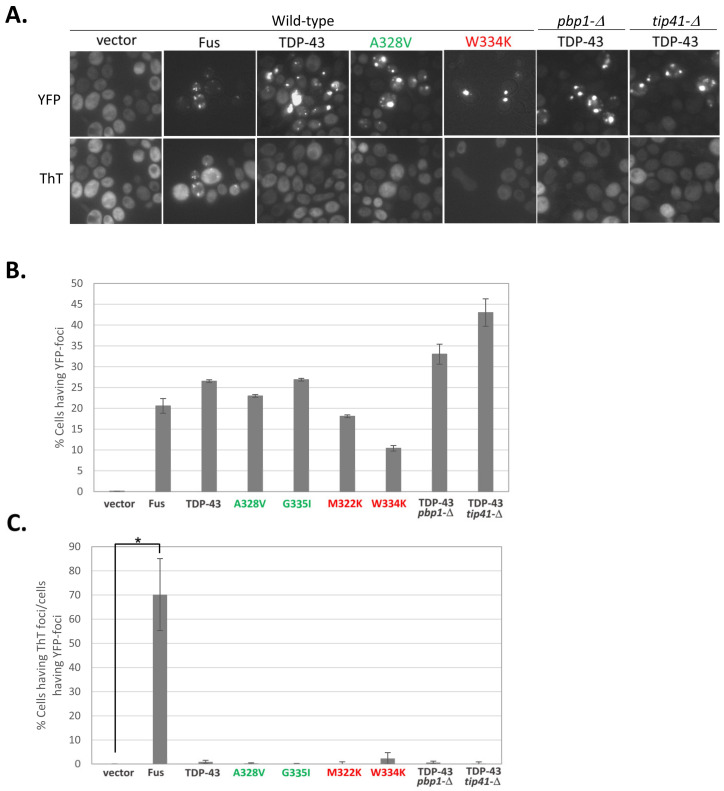
Foci formed by mutant TDP-43 are not stained with thioflavin T. Less toxic TDP-43 mutations are shown in green; more toxic mutations are shown in red. (**A**) Foci formed by wild-type or mutant TDP-43 are not stained with thioflavin T. All photos are the same scale. [*PIN*^+^] cells overexpressing wild-type TDP-43-YFP, the indicated mutant TDP-43-YFP, or FUS-YFP were grown for 16 h on solid synthetic medium with 2% galactose as the sole carbon source and then were stained with thioflavin T. FUS-YFP was used as a positive control for Thioflavin T staining. (**B**) Graph shows that cells still have YFP foci after thioflavin T staining. Three transformants for each were tested and averaged. (**C**) Thioflavin T only stained aggregates formed by FUS but not TDP-43 or its mutants. * indicates *p* < 0.05 in a paired two-tailed *t*-test with vector control.

As seen previously [7,35], cells transformed with YFP (Yellow Fluorescent Protein) tagged FUS or TDP-43 form fluorescent foci visible with a YFP filter. Only the FUS aggregates stain with thioflavin T, as seen with a CFP filter (Figure 1). Thus, wild-type TDP-43 aggregates are not amyloid. We also determined whether the larger more stable TDP-43 aggregates formed by mutations that reduce TDP-43 toxicity, e.g., the mutation with the alanine at position 328 changed to valine (A328V) or the glycine at position 335 changed to isoleucine (G335I), were amyloid. To do this, we attempted to stain cells expressing these mutant alleles with thioflavin T. Thioflavin T did not stain these aggregates, so it appears that they also do not form amyloids. Likewise, thioflavin T did not stain foci formed by wild-type TDP-43 in yeast with extragenic deletions (*pbp1*-*Δ* and *tip41*-*Δ*) that reduce TDP-43 toxicity, nor small unstable TDP-43 foci near nuclei formed by TDP-43 mutants that enhanced toxicity, e.g., the mutation with the tryptophan at position 334 changed to lysine (W334K) or the methionine at position 322 changed to lysine (M322K), were not amyloid (Figure 1).

**Figure 2:**
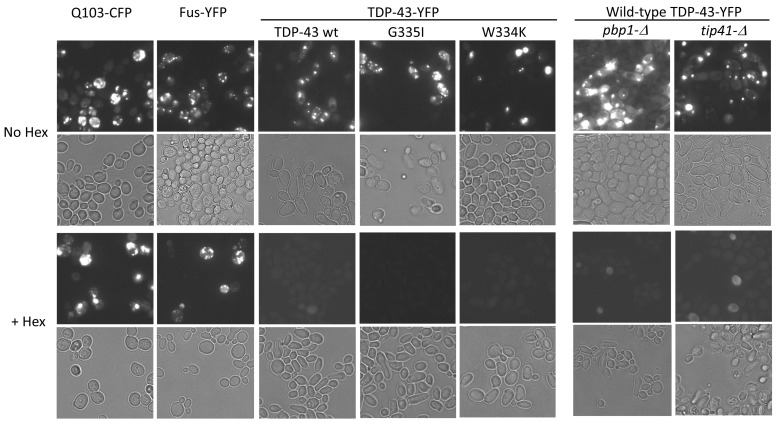
1,6-hexanediol dissolves TDP-43 foci. [*PIN*^+^] BY4741 and isogenic strains with *pbp1-Δ* or *tip41-Δ* were transformed with wild-type (wt TDP-43-YFP) or mutant TDP-43-YFP (G335I—less toxic or W334K—more toxic), or Fus-YFP. Q103-CFP was transformed into [*PIN*^+^] BY4741. These transformants all formed foci after TDP-43, FUS, or Q103 expression was induced on galactose medium. Cells with foci were incubated with (+Hex) or without (No Hex) 10% 1,6-hexanediol for 5 min. Q103-CFP and Fus-YFP foci were used as negative controls for 1,6-hexanediol-sensitive aggregates. More than 6 transformants of each type were examined. Fluorescent and bright-field images are shown. All images are at the same magnification scale.

To determine whether any TDP-43 aggregates in yeast are liquid-like, the cells were treated with 1,6-hexanediol, which preferentially dissolves liquid-like foci [36,37]. As reported previously [31], less toxic vs. more toxic TDP-43 mutants were, respectively, more frequently found in the cytoplasm vs. the nuclear periphery. 1,6-hexanediol dissolved all TDP-43 foci irrespective of location (Figure 2). This included TDP-43 aggregates formed with wild-type TDP-43, with intragenic TDP-43 mutations that enhance or reduce TDP-43 toxicity, or TDP-43 aggregates formed in the presence of *pbp1-Δ* or *tip41-Δ*, which reduce TDP-43 toxicity. This established that TDP-43 aggregates are liquid-like. The evidence does not support the hypothesis [31] that TDP-43 foci formed by less toxic TDP-43 mutants are amyloid while TDP-43 foci formed by more toxic TDP-43 mutants are liquid-like.

### 2.2. Effects of Enhancing TDP-43 Toxicity on Autophagy

**Figure 3:**
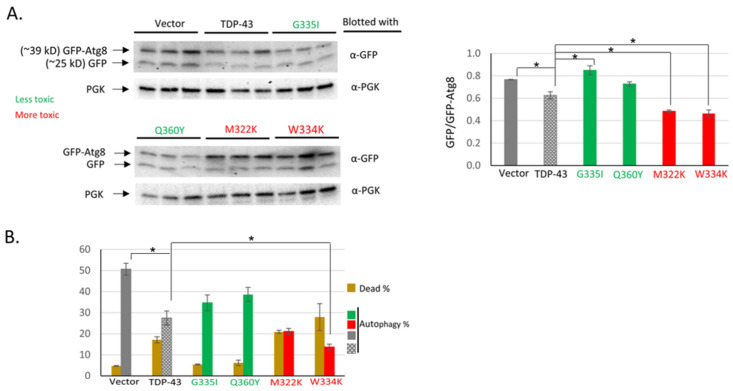
Enhancing TDP-43 toxicity with intragenic mutations reduces autophagy. BY4741 was double transformed with the copper-inducible *pCUP1-GFP-ATG8* reporter to measure autophagy, and plasmids expressing the *GAL1* promoter-driven wild-type (stippled grey), less toxic (green), or more toxic TDP-43 (red) or no TDP-43 (vector, solid grey). These were, respectively, p*GAL1-TDP-43* (TDP-43); p*GAL1-TDP-43 G335I* or p*GAL1-TDP-43 Q360Y*; p*GAL1-TDP-43 M322K or* p*GAL1-TDP-43 W334K*; and the vector (p*GAL1)*. (**A**) Immunoblot assay for autophagy. Autophagy is measured as the ratio of cleaved GFP to uncleaved GFP-ATG8 imaged with anti-GFP. PGK1 (anti-PGK, yeast 3-phosphoglycerate kinase) was used as an internal loading control. The graph shows the average and SEM of an image J analysis of the ratio of the density of GFP to GFP-ATG8 bands in gels from three independent transformants. * indicates *p* < 0.05 using a paired two-tailed *t*-test. (**B**) Microscopic assay for toxicity and autophagy. About 300–700 cells were scored for each transformant. The percentage of live cells showing autophagy is shown. Error bars represent the SEM calculated from three independent transformants. * indicates *p* < 0.05 in a paired two-tailed *t*-test.

As seen previously [32], the overexpression of wild-type TDP-43 significantly reduced autophagy relative to the vector control. Using GFP-ATG8 (Green Fluorescent Protein fused to autophagy-related protein 8) immunoblotting to measure autophagy showed that the expression of the more toxic TDP-43 mutants M322K and W334K reduced autophagy even more than wild-type TDP-43. In contrast, the expression of the TDP-43 mutants G335I and Q360Y with reduced toxicity had no effect on autophagy compared to the vector control (Figure 3A).

Similar results were obtained when autophagy was measured by examining the location of GFP-ATG8 in the cell. The more toxic TDP-43 mutant W334K, but not the less toxic G335I and Q360Y mutants, significantly reduced autophagy compared to wild-type TDP-43 (Figure 3B).

Finding that more toxic TDP-43 mutants reduced autophagy more than wild-type TDP-43 complements earlier data [32], which show that the *pbp1-Δ* and *tip41-Δ* modifiers, which reduce TDP-43 toxicity, restore autophagy. Furthermore, under these same conditions, but when TDP-43 was not overexpressed, *pbp1-Δ* and *tip41-Δ* had no effect on autophagy [32]. This suggests that TDP-43’s inhibition of autophagy causes a proportional level of toxicity.

### 2.3. Effects of TDP-43 and Extragenetic Modifiers or Mutations in TDP-43 on TOROID Formation

**Figure 4:**
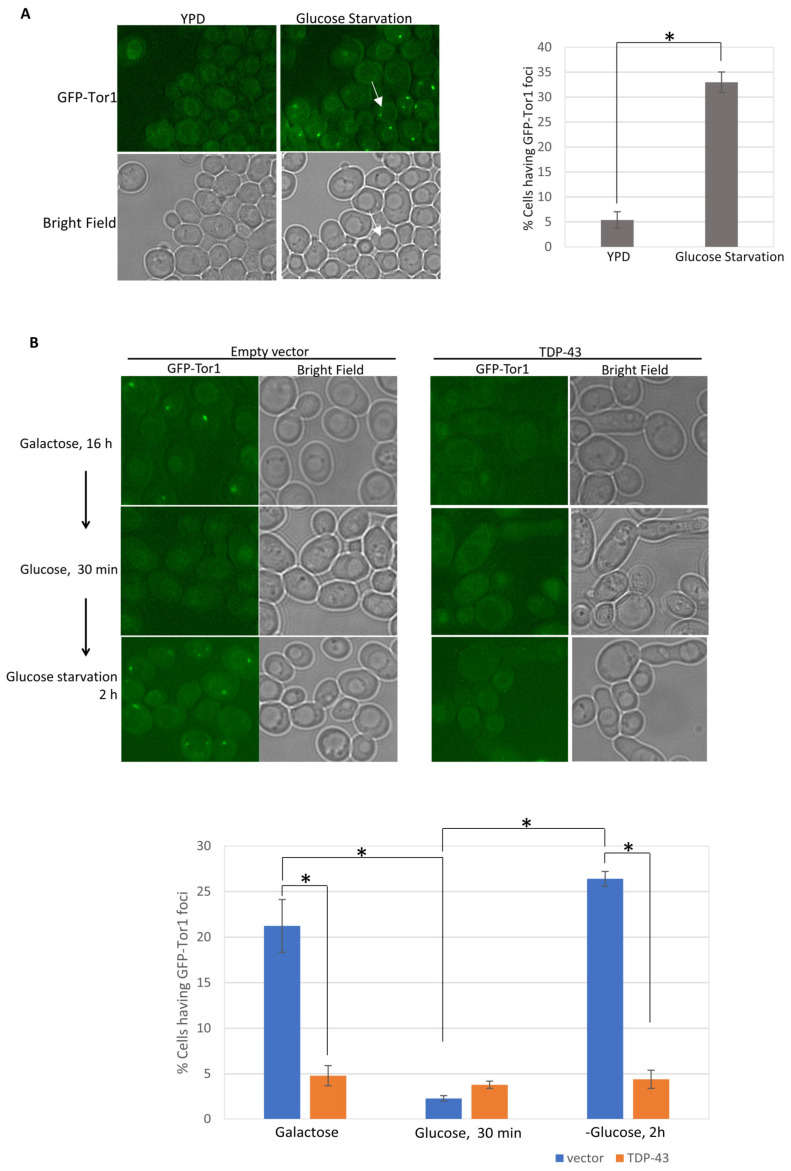
TDP-43 inhibits TOROID formation on galactose or caused by glucose starvation. In the absence of TOROIDs, integrated GFP-TOR1 (green fluorescent protein-tagged target of rapamycin) is diffuse in the cytoplasm and can be seen in the vacuolar membrane [34]. TOROIDs appear as GFP-TOR1 foci on the vacuole membrane [34]. Dead cells auto fluoresced under the DsRed and GFP filters and were excluded from the calculations. (**A**) Glucose starvation induces TOROID formation in the absence TDP-43 expression. Cells expressing GFP-TOR1 were examined with the GFP filter of a Nikon fluorescence microscope (photographed with a 100×/1.2 oil objective with 1.3 s exposure), before and after glucose starvation. White arrows point to an example of a vacuole in the bright field and the corresponding appearance of GFP-TOR fluorescence in that vacuolar membrane with TOROID focus on the membrane. The number of live cells with GFP-TOR1 foci were counted and averaged from about 500 cells from three independent subclones. Error bars represent the SEM calculated from three independent subclones. * indicates *p* < 0.05 in a paired two-tailed *t*-test. (**B**) The presence of TDP-43 reduces TOROID formation on galactose or under glucose starvation. Cells transformed with an empty vector (pGAL1) or TDP-43-expressing vector (pGAL1-TDP-43) and expressing GFP-TOR1 were grown in galactose to allow for the expression of TDP-43. TOROIDs appeared only in control cells without TDP-43. Subsequent growth on glucose for 30 min caused the galactose-induced TOROIDs to disappear. Following 2 h of glucose starvation, TOROIDs reappeared in cells without TDP-43, but TOROIDs did not appear in cells with TDP-43. The number of live cells with GFP-TOR1 cytoplasmic foci were averaged from about 500 cells of three independent transformants. Error bars represent the SEM calculated from three independent transformants. * indicates *p* < 0.05 in a paired two-tailed *t*-test. All photographs in A are at same magnification. Photographs in B are enlarged relative to A but all photographs in B are at the same magnification.

One hypothesis to explain the above results is that toxic TDP-43 prevents TORC1 from forming a TOROID structure, thereby leaving TORC1 available to inhibit autophagy. To investigate this, we tested the effects of wild-type, more toxic, and less toxic TDP-43 mutants on TOROID formation, as well as of extragenic modifiers that reduce TDP-43 toxicity. Glucose starvation was previously reported [34] to induce the formation of TOROIDS, which were measured by examining the location of the TORC1 subunit, TOR1 (target of rapamycin) tagged with GFP (GFP-TOR1). Without glucose starvation, TOR1-GFP was diffuse in the cytoplasm and could be seen in the vacuolar membrane, while only rare cells showed large foci. After glucose starvation, many cells had vacuole-associated TOR1-GFP foci indicative of TOROID formation [34]. We confirmed this result in our strains (Figure 4A).

To determine whether the expression of TDP-43 affects TOROID formation, cells with GFP-tagged TOR1 were transformed with a plasmid expressing TDP-43 under the *GAL1* promoter or a control empty vector, and transformants were grown in 2% galactose for 16 h to allow for the expression of TDP-43. Over 20% of cells not expressing TDP-43 showed TOROID foci, while less than 5% of cells expressing TDP-43 had foci (Figure 4B). When the cells with the plasmid expressing TDP-43 and the culture with the control empty vector were grown in galactose for 16 h, they were each transferred to plasmid-selective glucose medium and grown for 30 min, and the number of cells with an empty vector that contained foci was reduced 10-fold. However, growth in glucose did not affect the frequency of cells with foci (which was already very low) in the culture containing the TDP-43 plasmid. Then, when cells were transferred to glucose starvation medium for 2 h, foci reappeared in cells without TDP-43, but not in cells with TDP-43. Thus, TDP-43 inhibits TOROID formation induced by growth on galactose or by glucose starvation.

**Figure 5:**
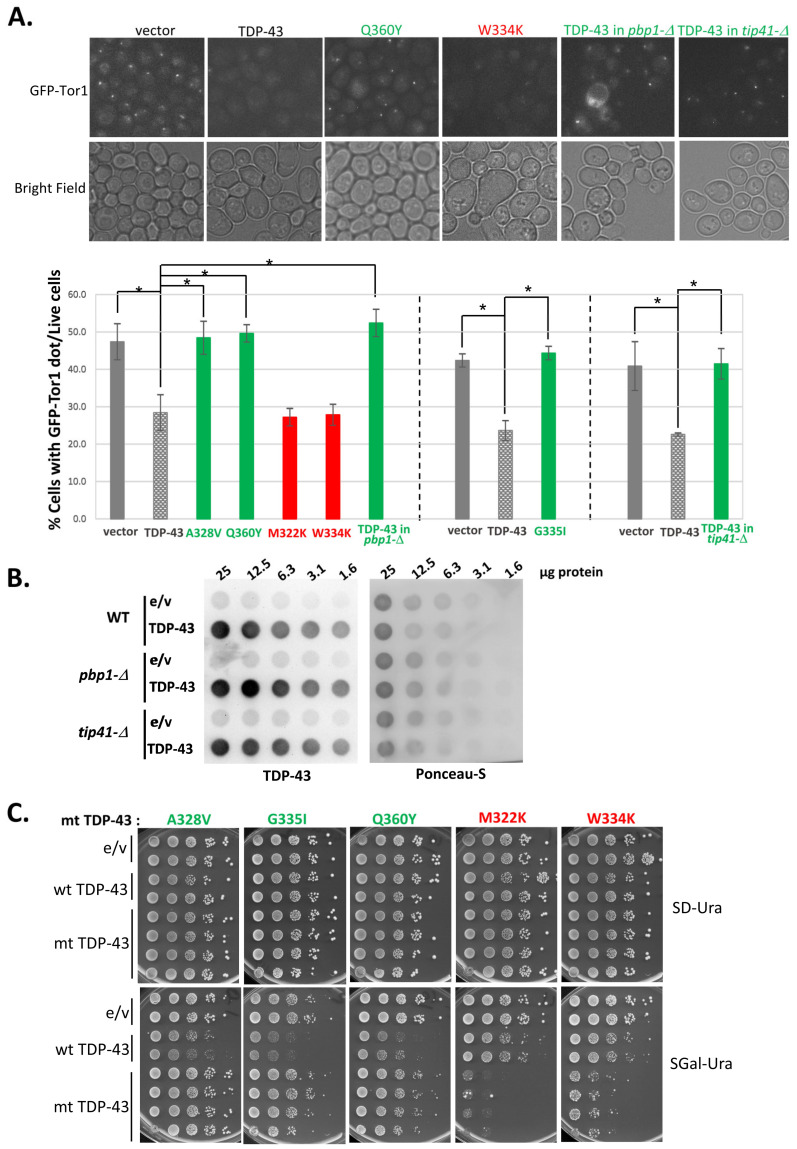
Toxicity of TDP-43 parallels effects on TOROID formation. GFP-TOR1 aggregation [34] is reduced by expression of TDP-43 and more toxic TDP-43 mutants but not by expression of less toxic TDP-43 mutants or wild-type TDP-43 in *pbp1-Δ* or *tip41-Δ* strains. (**A**) The percentage of cells with a GFP-TOR1 dot among live cells expressing various TDP-43 mutants or with extragenic modifiers that alter TDP-43 toxicity. Top panel shows GFP-TOR1 aggregation in sample cells with wild-type (TDP-43), toxic (written in red), and less toxic (written in green) TDP-43 mutant expression. The data are quantified in the graph below. Cells were stained with 0.5% trypan blue to exclude dead cells. Live cells with GFP-TOR1 cytoplasmic foci were counted and averaged from 3 independent transformants by examining about 500 cells per transformant. Error bars represent the SEMs calculated from three independent transformants. * indicates *p* < 0.05 in a paired two-tailed *t*-test. (**B**) Immuno-dot-blot assay of TDP-43 expression in the presence of the *pbp1-Δ* and *tip41-Δ* deletions that reduce TDP-43 toxicity. Whole cell lysates were prepared from cells grown in SGal-Ura medium for 16 h, serially diluted two-fold, and loaded to a PVDF (polyvinylidene difluoirde) membrane. The level of TDP-43 was measured with anti-TDP-43. Ponceau-S staining shows the total protein amount in each lysate. (**C**) Growth of cells expressing wild-type or mutant TDP-43. The mt TDP-43 (mutant TDP-43) shown is indicated on the top of each column. Mutations written in green were reported to reduce TDP-43 toxicity, while those in red enhanced toxicity [31]. Normalized serially diluted cells were spotted on plasmid-selective 2% glucose (SD-Ura) or 2% galactose (SGal-Ura) medium. Plates were scanned after 4 days of incubation at 30 °C.

We used growth on galactose to examine the effects of TDP-43 mutations on TOROID formation. Transformants with vector control, or plasmids expressing wild-type TDP-43 or mutants under the control of *GAL1* grown in plasmid-selective glucose medium, were transferred to synthetic galactose medium and grown for 16 h. In the absence of TDP-43, this induced the frequent formation of a single GFP-TOR1 aggregate per cell, indicative of TOROID formation. However, the overexpression of TDP-43 significantly reduced the fraction of cells with such aggregates (Figure 5A). Furthermore, TDP-43 mutations that reduced (A328V, Q360Y, G335I), but not those that enhanced (M322K, W334K), TDP-43 toxicity restored GFP-TOR1 aggregation (Figure 5A). The data in Figure 5C confirm that the TDP-43 mutants expressed in our experiments had the previously expected [31] effects on toxicity. Likewise, when either *TIP41* or *PBP1* was deleted, which reduced TDP-43 toxicity [19,32], TDP-43 expression no longer affected TOROID formation (Figure 5A). We used a dot-blot assay to confirm that the isogenic wild-type, *pbp1-Δ*, and *tip41-Δ* strains expressed TDP-43 at equal levels (Figure 5B). In summary, the expression of TDP-43 reduces TOROID formation, but extragenetic modifiers or mutations in TDP-43 that reduce its toxicity prevent this.

### 2.4. Both More Toxic and Less Toxic TDP-43 Bind to TORC1

**Figure 6:**
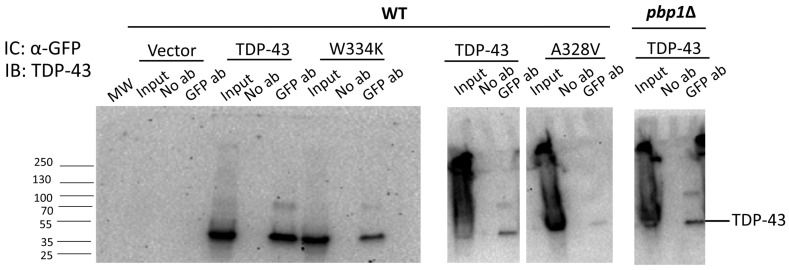
TDP-43 immunoprecipitates with GFP-TOR1. BY4741 with integrated TOR1 tagged with GFP (GFP-TOR1) [34] and its isogenic *pbp1Δ* derivative were transformed with the empty vector, TDP-43 WT, TDP-43 W334K (more toxic), and TDP-43 A328V (less toxic) (*GAL*, *URA3*, *CEN*). Lysates (Input) of cells grown in galactose medium to induce TDP-43 were immunoprecipitated with (GFP ab) or without (No ab) GFP antibodies, which pulled out GFP-TOR1. Proteins in the input and immunoprecipitates were separated on protein gels and blotted with TDP-43 antibody.

One hypothesis to explain the above results is that TDP-43 only binds to TORC1 when it inhibits TOROID formation, leaving TORC1 available to inhibit autophagy. To investigate this, we tested the ability of wild-type, more toxic, and less toxic TDP-43 mutants to bind to TOR1. To our surprise, TDP-43 encoded by all the alleles bound to TOR1 (Figure 6). Likewise, TDP-43 bound to TOR1 even in the presence of the *pbp1Δ* extragenic modifier that reduces both TDP-43’s toxicity and its effect on TOROID formation. Thus, the binding of TDP-43 to TORC1 is not sufficient to affect TORID formation or toxicity.

## 3. Discussion

Toxic aggregating proteins form amyloid as well as liquid-like aggregates. There is debate over which type of aggregate is toxic. The yeast Sup35 (translation termination factor eRF3) prion protein forms liquid-like aggregates under stress that are not toxic but instead promote yeast survival [38]. In contrast, the separation of cells containing liquid-like droplets from cells containing Sup35 amyloid foci on the basis of Dam-FRET (Distributed Amphifluoric Förster Resonance Energy Transfer) showed that the replication of cells containing the non-amyloid foci was inhibited relative to cells with amyloid condensates [39].

Aβ(Amyloid beta), implicated in Alzheimer’s disease, forms oligomers, as well as liquid-like aggregates that can mature into amyloid fibrils [40] and plaques. Oligomers are widely accepted to be more toxic than Aβ fibrils, plaques, or monomers. The type and structure of the toxic oligomers is still unknown [41].

Similarly, the toxic species of TDP-43 is unknown. Here, thioflavin T and 1,6-hexanediol were used to look for physical distinctions between large stable TDP-43 foci and smaller mobile TDP-43 foci. Previous studies showed that TDP-43 foci in yeast fail to stain with thioflavin T [2,35]. Since thioflavin T staining is indicative of amyloids, the current work investigated whether mutations that reduce TDP-43 toxicity and make foci more stable [31] allowed those foci to stain with thioflavin T. However, the stable foci with reduced toxicity did not stain with thioflavin T. This suggests that these structures are distinct from classical amyloid.

Although only foci formed by TDP-43 mutants that enhance toxicity are unstable and were suggested to be liquid-like [31], 1,6-hexanediol dissolved all TDP-43 foci, including those with reduced toxicity. Thus, the structures of the wild-type and mutant TDP-43 foci are all liquid-like.

Autophagy and its major regulator, mTORC1 (mammalian target of rapamycin complex 1), are implicated in several devastating neurodegenerative disorders based on genetic evidence. These disorders, which include Alzheimer’s disease (AD), Parkinson’s disease (PD), Huntington’s disease (HD), amyotrophic lateral sclerosis (ALS), and frontotemporal dementia (FTD), cause the progressive and permanent destruction of neurons, leading to cognitive and motor control problems. Autophagy is crucial for neuronal cells since they cannot divide to eliminate unwanted macromolecules or organelles. Furthermore, deleting essential autophagy genes in the brain can induce neurodegeneration, even without disease proteins, indicating autophagy as the core of neurodegenerative disease. As a result, for future therapeutic strategies, it will be helpful to develop a better understanding of how mTORC1 acts [42].

The work presented here shows that, in yeast, TDP-43’s toxicity and inhibition of autophagy parallels TDP-43’s effect on TOR1 (a component of TORC1). TOROIDs (TORC1 organized in inhibited domain) are large helical polymers of TORC1 that are associated with the vacuole. When TORC1 is not condensed in TOROIDs, it inhibits autophagy [33]. When TORC1 condenses into a TOROID, seen as a vacuolar-associated focus, its inhibition of autophagy is reduced [34]. By using tagged TOR1 as a reporter for TORC1, we showed that TDP-43 inhibited TORC1 from forming TOROIDs. Also, the modifiers *pbp1-Δ* and *tip41-Δ* and TDP-43 mutants that reduced TDP-43 toxicity, but not those that enhanced it, restored TOROID formation. In addition, mutations in TDP-43 that enhanced its toxicity, but not mutations that reduced TDP-43 toxicity, increased the reduction in autophagy. Thus, we propose that the reduction in the frequency with which TORC1 forms TOROIDS caused by wild-type TDP-43 or more toxic TDP-43 mutants allows unaggregated TORC1 to inhibit autophagy.

To assess whether TDP-43 binds to TORC1 and if this binding inhibits TOROID formation, we tested the ability of TORC1 to bind to wild-type TDP-43 as well as more toxic and less toxic mutant TDP-43. Surprisingly, not only did wild-type and more toxic TDP-43 bind to TORC1, but so did the less toxic TDP-43 allele that does not inhibit TOROID formation. Also, TDP-43 bound to TORC1 even in *pbp1-Δ* strains where TDP-43 does not inhibit TOROID formation. Thus, TDP-43 binding to TORC1 is not sufficient to inhibit TOROID formation. Instead, TDP-43’s effect on TOROID formation and toxicity may depend on the ability of the wild-type and more toxic alleles to enable other proteins to bind to a TORC1 interface, preventing TOROID formation. The less toxic TDP-43 allele and *pbp1-Δ* may not allow for this. Proteins binding to TORC1 have been shown to inhibit TOROID formation in yeast. The glucose-dependent activation of EGOC (complex of GTPases Gtr1 and Gtr2) causes the EGOC to bind to a TORC1 interface, which inhibits TORC1 from polymerizing into TOROID and therefore keeps TORC1 active. This leads to the inhibition of autophagy [43].

Another structure in mammalian cells also has some properties reminiscent of TOROIDs. This is the TOR–autophagy spatial coupling compartment (TASCC), where mTOR accumulates in glomerular podocytes. TASCC may serve to sequester mTOR located on the trans side of Golgi. In yeast, the Rag GTPase-dependent movement of TORC1 from around vacuoles to single foci may be a mechanism to regulate TORC1. Likewise, mTOR recruitment to the TASCC is amino acid- and Rag guanosine triphosphatase-dependent [44].

## 4. Materials and Methods

### 4.1. Strains and Plasmids

The strain BY4741 ([*PIN*^+^] *MATa his3Δ1 leu2Δ0 met15Δ0 ura3Δ0*) was used to measure TDP-43 toxicity and its effect on autophagy. To measure TOROID formation and assess the immunoprecipitation of TOR1, strain SKY222, which Dr. Robbie Joséph Loewith [34] kindly provided, was used. SKY222 was constructed by integrating N-terminally tagged Tor1 (GFP-TOR1) controlled by its native promoter into BY4741 [45]. This made the strain *LEU2*. *PBP1* and *TIP41* were disrupted in SKY222 using library deletion strains of *PBP1* and *TIP41* in BY4741 obtained from Open Biosystems(Huntsville, AL, USA, Cat # YSC1053). The deletion cassettes from these strains were amplified using upstream and downstream gene-specific 45 mer primers [46], and the amplicons were transformed into SKY222 by selecting for G418-resistant transformants. All disruptions were confirmed with PCR (Polymerase Chain Reaction). Site-directed mutagenesis was used to make plasmids that express untagged TDP-43 and its mutants under control of the *GAL1* promoter. TDP-43 mutations that enhance (M322K, W334K) or reduce (A328V, G335I, Q360Y) TDP-43 toxicity [31] in TDP-43 were inserted in the pDONR221-TDP43 gateway clone (p2273) with a stop codon [47] using the QuickChange II XL Site-Directed Mutagenesis Kit (Agilent, Cedar Creek, TX, USA, Cat # 200521). All constructs were confirmed by sequencing. These TDP-43 entry plasmids were used with destination vectors p2129 (pAG416GAL1-ccdB, *URA3*, *CEN*, Addgene https://www.addgene.org plasmid #14147) and p2245 (pAG415GAL1-ccdB, *LEU2*, *CEN,* Addgene plasmid #14145), deposited by Susan Lindquist [48], to construct, respectively *GAL1, CEN, URA3* plasmids, pAG416 GAL1 TDP43 (wt, p2665; A328V, p2706; G335I, p2707; Q360Y, p2708; M322K, p2709; W334K, p2710) or *GAL1, CEN, LEU2* plasmids, pAG415 GAL1 TDP43 (wt, p2368; G335I, p2701; Q360Y, p2702; M322K, p2703; W334K, p2704). Plasmids pAG416 GAL1-ccdB and pAG415 GAL1-ccdB, respectively, served as the vector controls for the pAG416, GAL1, TDP43 and pAG415, GAL1, TDP43 series. The plasmid pGFP-ATG8 (alias p2571 *URA3, CEN*, Addgene #49423 deposited by Daniel Klionsky was used to measure autophagy. *GFP-ATG8* was expressed from the *CUP1* promoter.

### 4.2. Reagents

Thioflavin T, digitonin, triton X-100, lithium acetate dihydrate, polyethylene glycol 3350, deoxyribonucleic acid sodium salt from salmon testes (ss-DNA), glycerol, galactose, yeast nitrogen base without amino acids, amino acids, uracil, and adenine were purchased from Sigma (St. Louis, MO, USA); dextrose from Fisher Scientific (Fair Lawn, NJ, USA); 1,6-hexanediol from EMD Millipore (Darmstadt, Germany); phenylmethylsulfonyl fluoride (PMSF), dithiothreitol (DTT), and ammonium sulfate from Thermo Fisher Scientific (Rockford, IL, USA); the EconoTaq PLUS 2X Master Mix from Lucigen (Middleton, WI, USA); FM4-64 from Invitrogen (Eugene, OR, USA); mini-PROTEAN TGX gels from Bio-Rad (Hercules, CA, USA); and yeast extract, peptone, bacto-tryptone, and agar from Apex (El Cajon, CA, USA).

### 4.3. Transformation

Plasmids were transformed into yeast using the lithium acetate method [49]. Briefly, after adding 0.2 μg of plasmid DNA and 0.5 μg ss*-*DNA to 100 μL of yeast competent cells, 300 μL of transformation solution containing 50% (*w*/*v*) polyethyene glycol 3550, 0.1 M lithium acetate, and 0.5 μg ss*-*DNA was added before heat shocking the cells at 42 °C for 8 min. They were then plated on plasmid-selective glucose medium.

### 4.4. Scoring for Growth

Cells were grown in standard yeast medium [50]. Following overnight growth in plasmid-selective glucose medium, cells were normalized and 10× serial dilutions in water were spotted on plasmid-selective 2% glucose or 2% galactose medium. Plates were scanned after 4 days of incubation at 30 °C.

### 4.5. Microscopy

Cells were imaged with a Nikon Eclipse E600 (Tokyo, Japan) fluorescent microscope with Nikon 100×/1.23na or 60×/1.4na oil objectives.

### 4.6. Staining with Thioflavin T

Cells were stained with thioflavin T as described previously [2]. Thioflavin T staining was detected by fluorescence with a CFP filter. Cells were observed in the bright field.

### 4.7. Dissolving Liquid-like Foci with 1,6-Hexanediol

Hexanediol experiments were conducted as previously described [51]. Cells transformed with plasmids encoding wild-type or mutant TDP-43-YFP were cultured overnight in glucose medium; then, TDP-43-YFP was overexpressed in plasmid-selective galactose medium for an additional 4 h, after which the cells were incubated with 2 mg/mL digitonin, with or without 10% 1,6-hexanediol, for 5 min before being photographed with YFP filters.

### 4.8. Measuring TOROID Formation

To examine the effect of glucose starvation on TOROID formation, BY4741 cells with integrated GFP-TOR1 grown in complex glucose medium (YPD) were washed once and transferred to a synthetic medium lacking glucose, where they were grown for an additional 2 h. The frequency of live cells with GFP-TOR1 aggregates was determined microscopically in three subclone samples, each consisting of approximately 500 cells, using the GFP filter of the fluorescence microscope with a 1.3 s exposure (Nikon 100×/1.23 oil objective). A similar method was used to examine the effects of TDP-43 on glucose starvation-induced TOROID formation, except that cells transformed with *GAL1*, *CEN*, *URA3* pAG416GAL1-TDP43, or its empty vector were used and were grown on galactose for 16 h to allow for the expression of TDP-43 before being washed and grown on plasmid-selective glucose medium for 30 min, followed by washing and transfer to a medium lacking glucose as described above. Cells were examined with a DsRed filter, where autofluorescence indicated dead cells.

Also, the *GAL1*, *CEN*, *URA3* pAG416GAL1-TDP43 plasmid series was transformed into isogenic BY4741 cells with integrated GFP-TOR1 and either wild-type or *pbp1-Δ* and *tip41-Δ* deletions. Transformants selected on plasmid-selective glucose medium (SD-Ura) were grown overnight in liquid SD-Ura, washed, and resuspended in 5 mL plasmid-selective galactose medium (SGal-Ura) at OD_600_ = 1. They were then grown for another 16 h. Cells were stained with trypan blue to detect dead cells and photographed with a Nikon Eclipse E600 fluorescent microscope (Nikon 60×/1.4 oil objective) in the bright field (to see blue dead cells) and with a GFP filter to detect GFP-Tor1 foci. TOROID formation was measured as the fraction of live cells that had GFP foci.

### 4.9. Measuring Autophagy

Autophagy was scored (1) by determining the cellular location of GFP-ATG8 using fluorescent microscopy and (2) by determining the fraction of GFP-ATG8 that was cleaved using Western blots. Untagged TDP-43 was expressed from the *GAL1*, *LEU2*, *CEN* plasmid series, *pAG415GAL1-TDP-43.* The methods were as described previously [32] except that for the microscopy procedure, cells were grown on plasmid-selective 2% galactose plates instead of in liquid medium. Freshly grown cells from plasmid-selective dextrose plates were spread onto plasmid-selective medium with 2% galactose, 50 µM Cu^++^, and 1.6 nM FM4-64, to, respectively, induce the expression of TDP-43 or its mutants, induce GFP-ATG8, and stain vacuoles. Cells were then grown for 16 h at 30 °C. Live cells were scored for autophagy by counting the cellular location of GFP-ATG8 fluorescence. As previously reported [32], cells with fluorescence concentrated in the vacuole without punctate cytoplasmic foci, or live cells with no fluorescence, were considered to have undergone autophagy. Cells with diffuse cytoplasmic fluorescence lacking vacuolar fluorescence, or those with cytoplasmic foci despite fluorescence in the vacuole, were scored as lacking autophagy. Dead cells were excluded in the analysis. The % of dead cells was calculated as the ratio of dead trypan blue-stained cells to total stained and unstained cells identified in the bright field. FM4-64-stained vacuoles and GFP-ATG8 were, respectively, detected with mCh and GFP filters.

### 4.10. Co-Immunoprecipitation of TOR1-GFP and TDP-43

SKY222, which is BY4741 with integrated TOR1 tagged with GFP (GFP-TOR1) [34], and its isogenic *pbp1Δ* derivative were transformed with an empty vector, TDP-43 WT, TDP-43 W334K (more toxic) and TDP-43 A328V (less toxic) (*GAL*, *URA3*, *CEN*). Transformants were selected and patched on plasmid-selective glucose medium and were checked for the presence of elongated cell shapes indicative of TDP-43 expression after growth on galactose medium. Transformants grown on plasmid-selective glucose medium overnight were washed and resuspended in plasmid-selective galactose medium for 24 h. Lysates were prepared as described previously with slight modifications [52]. Cells were resuspended in lysate buffer (50 mM Tris-HCl, pH 8.0, 150 mM NaCl, 10% glycerol, 1 mM DTT, 10 mM EDTA) supplemented with an EDTA-free protease inhibitor cocktail (Roche, Mannheim, Germany), 10 mM PMSF. Cell lysates were incubated for 10 min at 4 °C following the addition of Triton X-100 (0.2% final concentration), and then clarified by centrifugation for 10 min at 10,000× *g* at 4 °C. Protein concentrations were determined with a Bradford Assay using Protein Assay Dye Reagent Concentrate (Bio-Rad, Hercules, CA, USA).

For immunocapture, 800 μL of the precleared lysate containing 2.0 mg/mL proteins was incubated with 2 μL of GFP antibody (Roche, Mannheim, Germany) for 2 h on ice. Samples were then mixed with 50 μL magnetic beads with immobilized G protein (Miltenyi Biotec Inc., Auburn, CA, USA) and incubated on ice for 1 h. Nonspecifically bound proteins were washed off as described previously [47].

Co-immunoprecipitated proteins were eluted with hot sample buffer and were then analyzed by electrophoresis and immunoblotting with TDP-43 antibody (Proteintech Group, Rosemont, IL, USA). Total (input) protein was boiled at 95 °C for 5 min in 2% SDS sample buffer containing 80 mM DTT.

### 4.11. Statistical Methods

All data were analyzed with Microsoft Excel (Microsoft Office Professional Plus 2019) using a two-tailed Student’s *t* test to calculate the statistical significance of comparisons. *p* values of less than 0.05 were considered significant. At least three samples were analyzed for all experiments. The data from multiple experiments are presented as the standard error of the mean, SEM, generated by Microsoft Excel.

## 5. Conclusions

The data show that aggregates formed by TDP-43 in yeast have liquid-like rather than amyloid properties. This is true in the presence of TDP-43 extragenic modifiers or intragenic mutants that either reduce or enhance TDP-43 toxicity. Why some liquid-like TDP-43 aggregates are more toxic than others is unknown.

Also more toxic vs. less toxic TDP-43, respectively, had a greater or smaller inhibiting effect on TOROID formation and autophagy. This suggests that much of TDP-43’s toxicity comes from reducing the frequency with which the autophagy inhibitor, TORC1, forms TOROID structures where TORC1 cannot function. Because both TDP-43 alleles that do and do not prevent TOROID formation bind to TORC1, we hypothesize that TOROID-inhibiting but not non-TOROID-inhibiting TDP-43 enable other proteins to bind to a TORC1 interface, preventing TOROID formation.

So far, TOROID structures have only been seen in budding yeast [53]. However, structures of the TORC1 mammalian counterpart (mTORC1) and the yeast TOROID are consistent with the existence of TOROID in mammalian cells. Indeed, the overall yeast TORC1 structure extracted from TOROIDs is very similar to mTORC1 [43]. If the findings described here in yeast are verified in mammalian cells, drugs that prevent TDP-43 from inhibiting TOROID formation may prove therapeutic for a variety of neurodegenerative diseases characterized by TDP-43 aggregation.

## Data Availability

The data presented in this study are available in the article.
